# Supplementary material for: Antibodies targeting a quaternary site on SARS-CoV-2 spike glycoprotein prevent viral receptor engagement by conformational locking
Source: Immunity. 2023 Oct 10;56(10):2442–2455.e8. doi: 10.1016/j.immuni.2023.09.003 (PMC10588992; doi:10.1016/j.immuni.2023.09.003)
Supplement: Document S1. Figures S1–S6 and Tables S1 and S2 [file mmc1.pdf]

Immunity, Volume *56*

Supplemental information

Antibodies targeting a quaternary site on SARS-CoV-2 spike glycoprotein prevent viral receptor engagement by conformational locking

Lihong Liu, Ryan G. Casner, Yicheng Guo, Qian Wang, Sho Iketani, Jasper Fuk-Woo. Chan, Jian Yu, Bernadeta Dadonaite, Manoj S. Nair, Hiroshi Mohri, Eswar R. Reddem, Shuofeng Yuan, Vincent Kwok-Man Poon, Chris Chung-Sing Chan, Kwok-Yung Yuen, Zizhang Sheng, Yaoxing Huang, Jesse D. Bloom, Lawrence Shapiro, and David D. Ho

Table S1. Subject information. See also **Figure 1.**

Sample ID	Symptoms	Infection & vaccination history	Days between infection and 1st dose	Days between 1st dose and 2nd dose	Blood collection days post 2nd vaccination
Patient 12	Shortness of breath, fatigue, muscle pain, joint pain, fever headache, lack of taste, lack of smell, and confusion	R.1 (B.1.1.316.1)/ mRNA-1273/ mRNA-1273	20	28	7

Table S2. CryoEM data collection and model refinement. See also **Figure 2.**

	SARS-CoV-2 S2P + 12-16 Fab	SARS-CoV-2 S2P + 12-19 Fab	SARS-CoV-2 S2P + 4-33 Fab
**EMDB ID**	26583	26584	26964
**PDB ID**	7UKL	7UKM	8CSJ
**Data Collection**			
Microscope	FEI Titan Krios	FEI Titan Krios	FEI Titan Krios
Voltage (keV)	300	300	300
Magnification	105k	105k	81k
Defocus Range (μm)	-0.8/-2.0	-0.8/-2.0	-0.8/-2.0
Camera	Gatan K3 BioQuantum	Gatan K3 BioQuantum	Gatan K3 BioQuantum
Pixel Size (Å/pix)	0.83	0.83	1.07
Recording Mode	counting	counting	counting
Dose Rate (e^-/pixel/s)	16	16	16
Electron Dose (e^-/Å^2)	58	58	42
**Data Processing**			
Software	cryoSPARC v3.3	cryoSPARC v3.3	cryoSPARC v3.3
Micrographs used	3,691	4,042	6,100
Number of Particles	463,850	256,243	420,343
Symmetry	C1	C1	C3
Box Size (pix)	440	440	384
Global Map FSC0.143 (Å)	3.09	3.03	3.56
**Refinement and Validation**			
Software	Phenix, ISOLDE, Rosetta	Phenix, ISOLDE, Rosetta	Phenix, ISOLDE
Initial Model Used	6XM5(Spike), Alphafold(Fab)	6XM5(Spike), Alphafold(Fab)	7LAB(Spike), SAbPred(Fab)
Number of Atoms	30,234	30,145	32,073
Protein Residues	3,795	3,771	4,038
Ligands	NAG: 48	NAG: 47	NAG: 53
Model vs. Data CC (mask)	0.79	0.87	0.7
RMS deviations			
Bond lengths (Å) (# >4σ)	0	0	1
Bond angles (°) (# >4σ)	17	2	65
MolProbity Score	1.25	1.07	1.96
Clashscore (all atom)	2.42	1.41	3.57
Poor rotamers (%)	0	0	0
Ramachandran plot			
Favored (%)	96.55	96.84	92.95
Allowed (%)	3.45	3.16	7.05
Outliers (%)	0	0	0

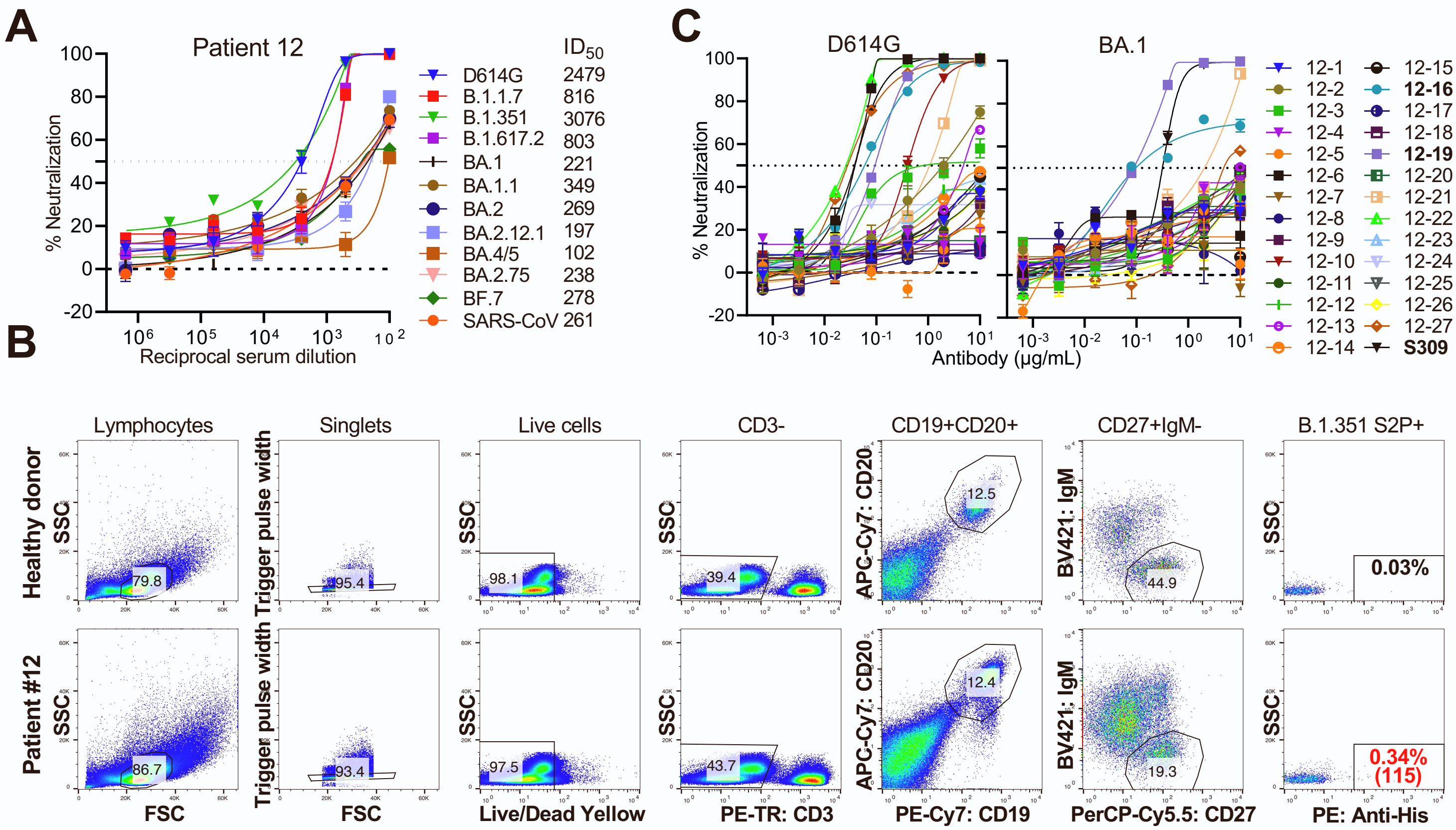

D

IC50 (μg/mL)	D614G	BA.1	IC50 (μg/mL)	D614G	BA.1	IC50 (μg/mL)	D614G	BA.1
12-1	>10	>10	12-10	0.396	>10	12-19	0.094	0.088
12-2	1.942	>10	12-11	>10	>10	12-20	>10	>10
12-3	0.860	>10	12-12	>10	>10	12-21	1.069	2.051
12-4	>10	>10	12-13	4.789	9.910	12-22	0.026	>10
12-5	>10	>10	12-14	>10	>10	12-23	>10	>10
12-6	0.038	>10	12-15	>10	>10	12-24	>10	>10
12-7	>10	>10	12-16	0.059	0.097	12-25	>10	>10
12-8	>10	>10	12-17	>10	>10	12-26	>10	>10
12-9	>10	>10	12-18	>10	>10	12-27	0.028	4.839
S309	0.039	0.318						

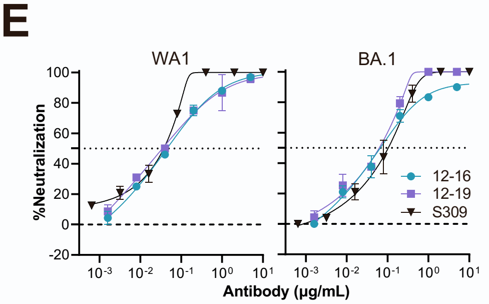

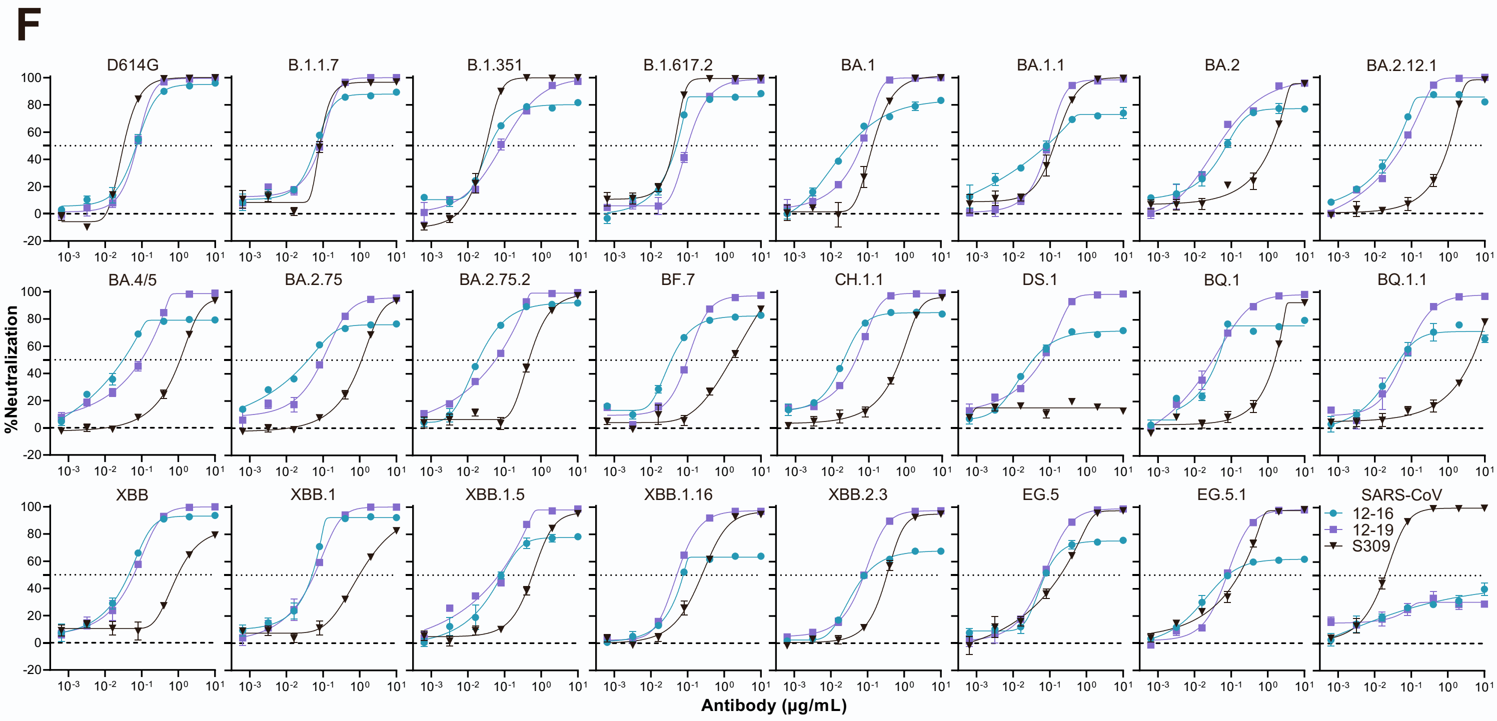

Figure S1. Neutralization potency of Patient 12 serum and the isolated mAbs.

(A) Neutralization curves and ID_{50} titers of the convalescent serum from Patient 12 against pseudotyped SARS-CoV-2 variants and SARS-CoV.

(B) Gating strategy for sorting of trimer-specific memory B cells by flow cytometry. The inset numbers indicate the percentage (and absolute number) of spike trimer-specific memory B cells obtained from healthy donor 1 and Patient 12.

(C) Neutralization curves of 27 isolated mAbs from Patient 12 against D614G and BA.1 pseudotyped viruses.

(D) Neutralization IC_{50} titers summarized from panel C.

(E) Neutralization curves of 12-16, 12-19, and S309 against authentic SARS-CoV-2 WA1 and BA.1.

(F) Neutralization curves of 12-16, 12-19, and S309 against both SARS-CoV-2 and SARS-CoV pseudoviruses.

Data are representative of those obtained in three independent experiments and shown as mean ± SEM.

A

mAbs	HV	HD	HJ	Isotype	H_SHM	CDRH3_len	HCDR3	LV	LJ	L_SHM	CDRL3_len	LCDR3
12-16	IGHV3-30*18	IGHD3-9*01	IGHJ6*02	IGHG3*04	1.70%	25	AKDLSFYYDISTGYYPQSYNSGTDV	IGLV3-1*01	IGLJ*01	1.40%	11	QGWDRSTGYYV
12-19	IGHV3-33*01	IGHD3-9*01	IGHJ6*02	IGHG1*04	2.70%	26	ARDRTPVYDILTGYYWPPPRVDGMDV	IGKV2-29*02	IGKJ2*02	2.30%	9	MQSIQLPFT

B

C

D

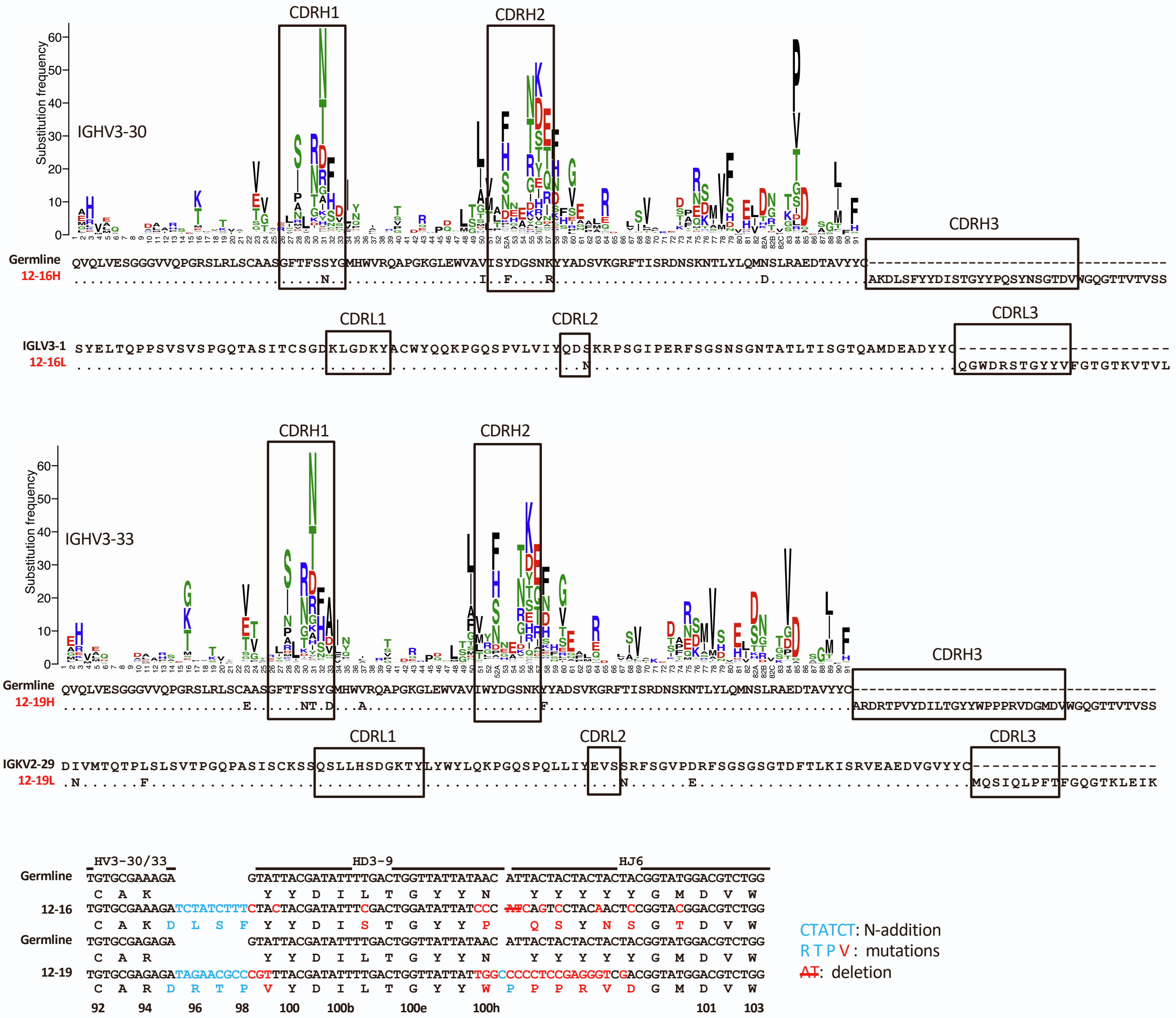

Figure S2. Genetic analysis for mAbs12-16 and 12-19.

(A) Germline gene assignment for mAbs 12-16 and 12-19.

(B) Gene-specific substitution profile (GSSP) for mAb 12-16.

(C) Gene-specific substitution profile (GSSP) for mAb 12-19. For (B) and (C), the dots represent the conserved residues in antibody sequence, as compared with the germline gene. The CDRs are highlighted by rectangles.

(D) CDRH3 VDJ junction analysis for mAbs 12-16 and 12-19. Germline nucleotides and amino acid residues are shown in black with the corresponding junctions colored in light blue. Somatic hypermutations are colored in red. Nucleotides deleted by exonuclease trimming are indicated with strikethrough. The blue nucleotides represent the N and P nucleotide additions at the junctions.

See also **Figure 1**.

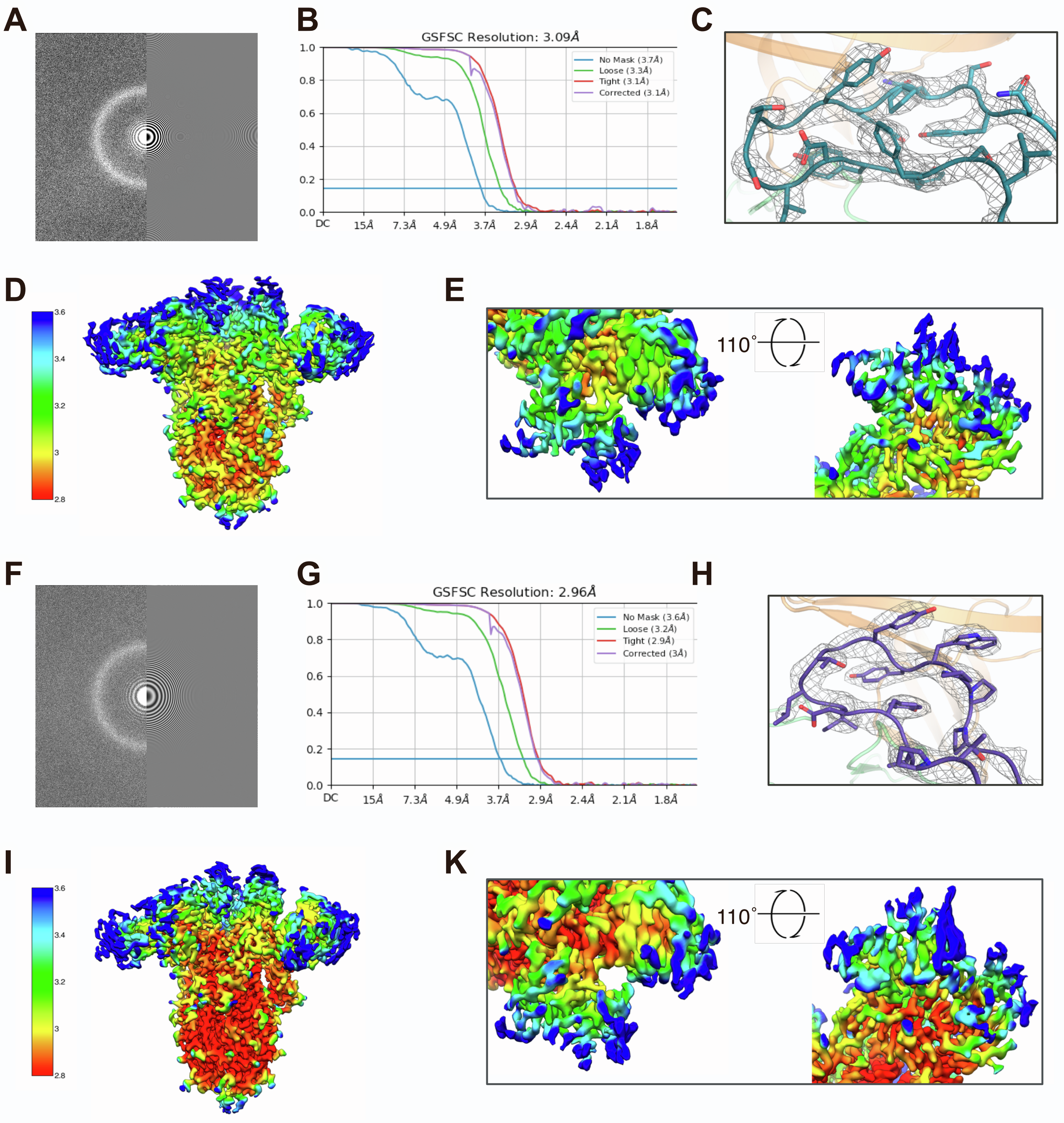

Figure S3. Cryo-EM data for mAbs 12-16 (A - E) or 12-19 (F - K) in complex with SARS-CoV-2 D614G spike trimer.

(A) and (F) Micrograph power spectrum (left) with contrast transfer function fit (right).

(B) and (G) Global refinement Fourier Shell Correlation curve showing overall resolution.

(C) and (H) Map density shown as mesh for mAb CDRH3 loop showing side chain fits. (D) and (I) Local resolution mapped onto global refinement reconstruction.

(E) and (K) Local resolution for the antibody interfaces, shown from top and bottom.

See also **Figure 2**.

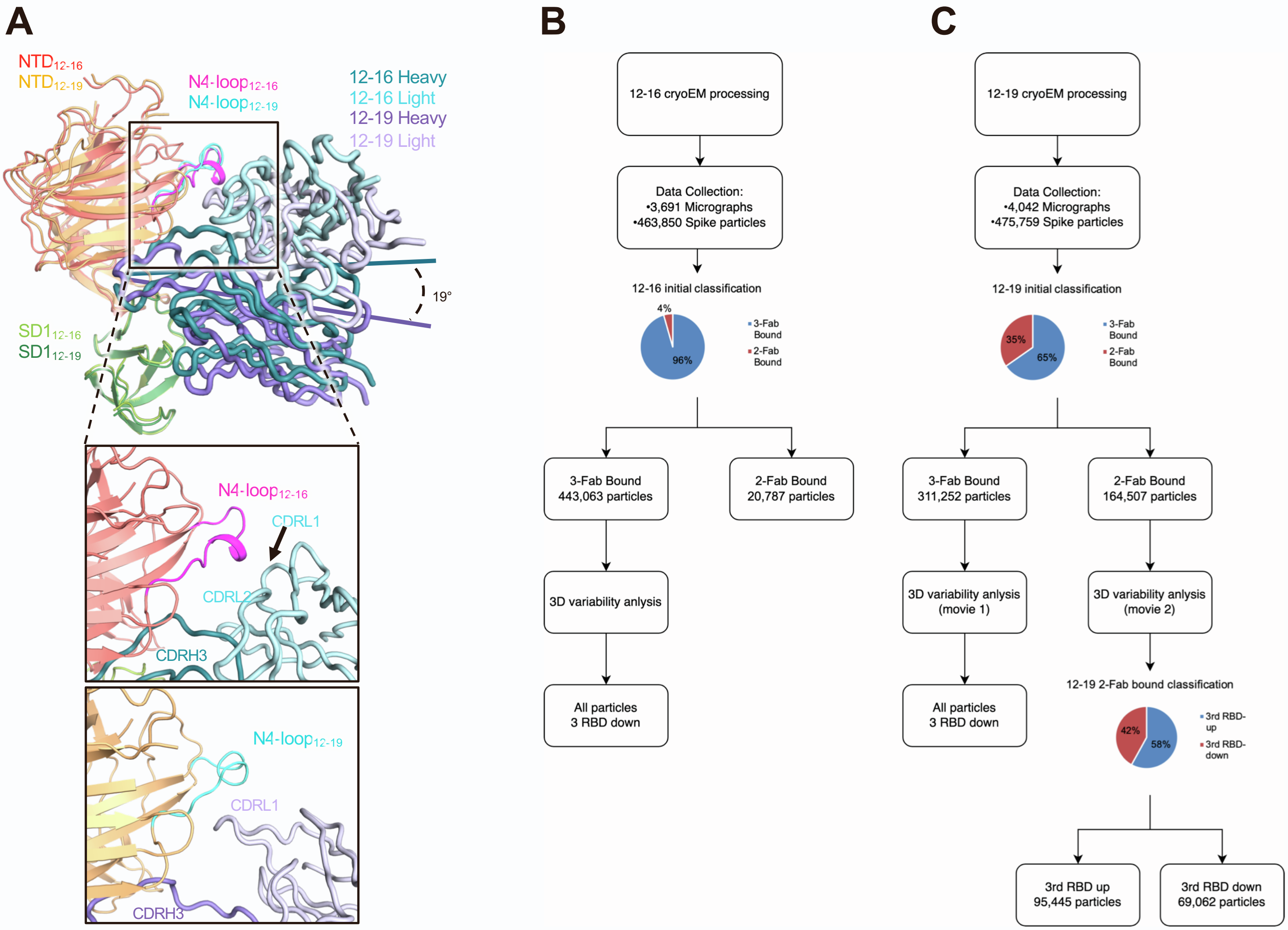

Figure S4. Structural comparison of mAbs 12-16 and 12-19 bound to SD1 and NTD, and cryo-EM processing and particle classification pipeline.

(A) The difference in angle of approach between heavy chains of 12-16 and 12-19 is shown. The close-up view highlights the CDR loop contacts between the antibody light chains and the N4-loop. See also **Figure 2**.

(B) For 12-16, nearly all spike particles were classified as 3-Fab-bound. See also **Figure 3**.

(C) For 12-19, spike particles were classified as 3- or 2-Fab-bound. See also **Figure 3**.

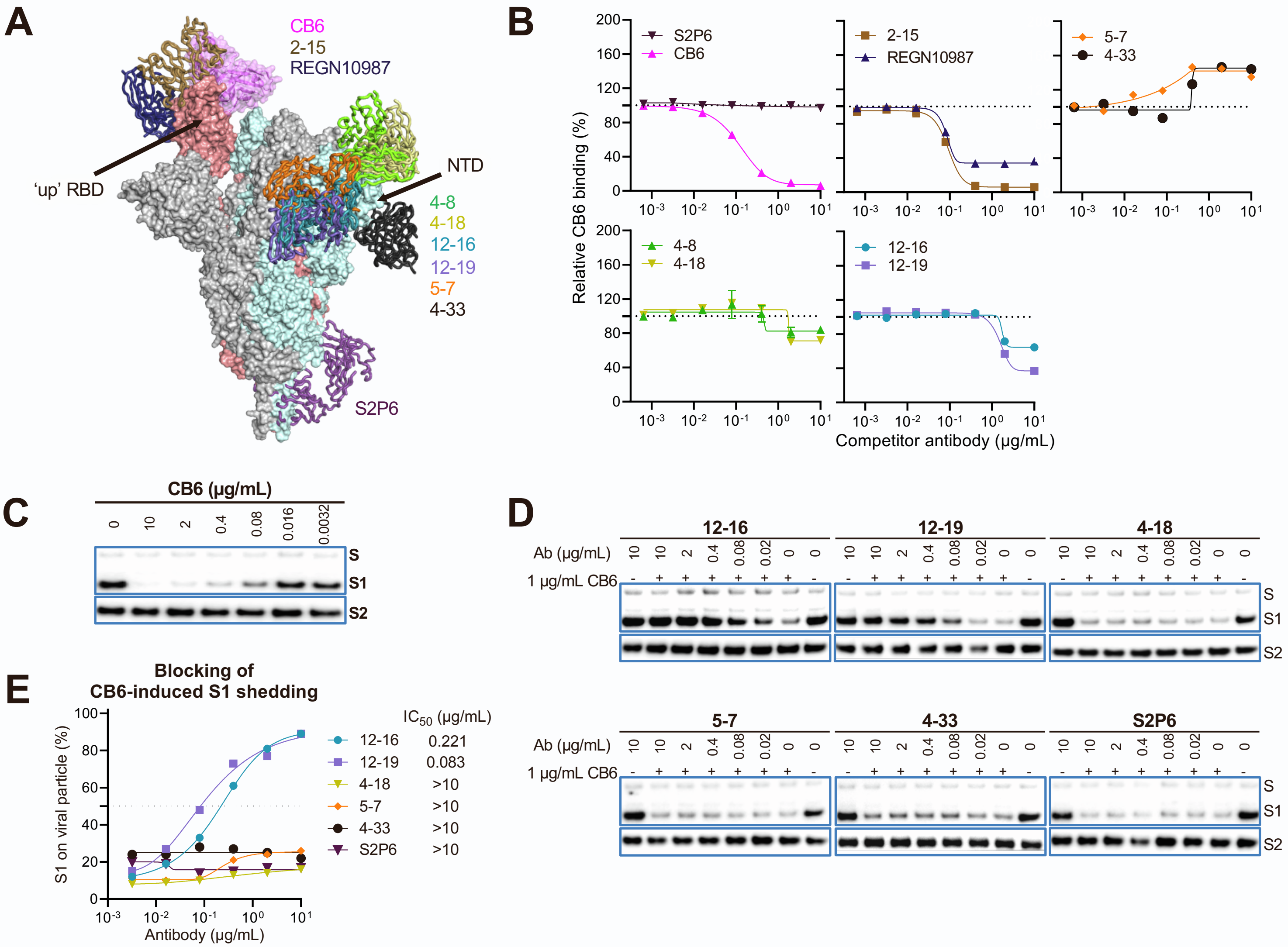

Figure S5. 12-16 and 12-19 inhibit CB6-spike binding and CB6-induced S1 shedding.

(A) Antibodies in complex with SARS-CoV-2 spike with one RBD in the up position (PDB: 7KRR). All of these antibodies can neutralize SARS-CoV-2 except 4-33. CB6 targets the RBD in the "up" position exclusively.

(B) Competition assay of CB6 binding to cell surface-expressed SARS-CoV-2 D614G spike in the presence of competitor antibodies. The data are shown as the mean ± SEM.

(C) CB6-induced S1 shedding from SARS-CoV-2 virions. D614G pseudovirus particles were incubated with CB6 at different doses for one hour at 37°C before the retained S1 and S2 subunits were determined by western blot.

(D) Inhibition of CB6-induced S1 shedding from the spike trimers on SARS-CoV-2 virions by the indicated antibodies. D614G pseudovirus particles were incubated with the indicated antibodies for one hour prior to incubating with 1 μg/mL CB6 for another one hour. The retained S1 and S2 subunits were determined by western blot.

(E) The intensities of the S1 and S2 glycoprotein bands in **(D)** were measured and the S1/S2 ratios are shown. Numbers denote the concentration of each antibody which inhibited half of the shedding of S1.

The results in **B**, **C**, **D, and E** are representative of those obtained in two independent experiments. See also **Figure 4**.

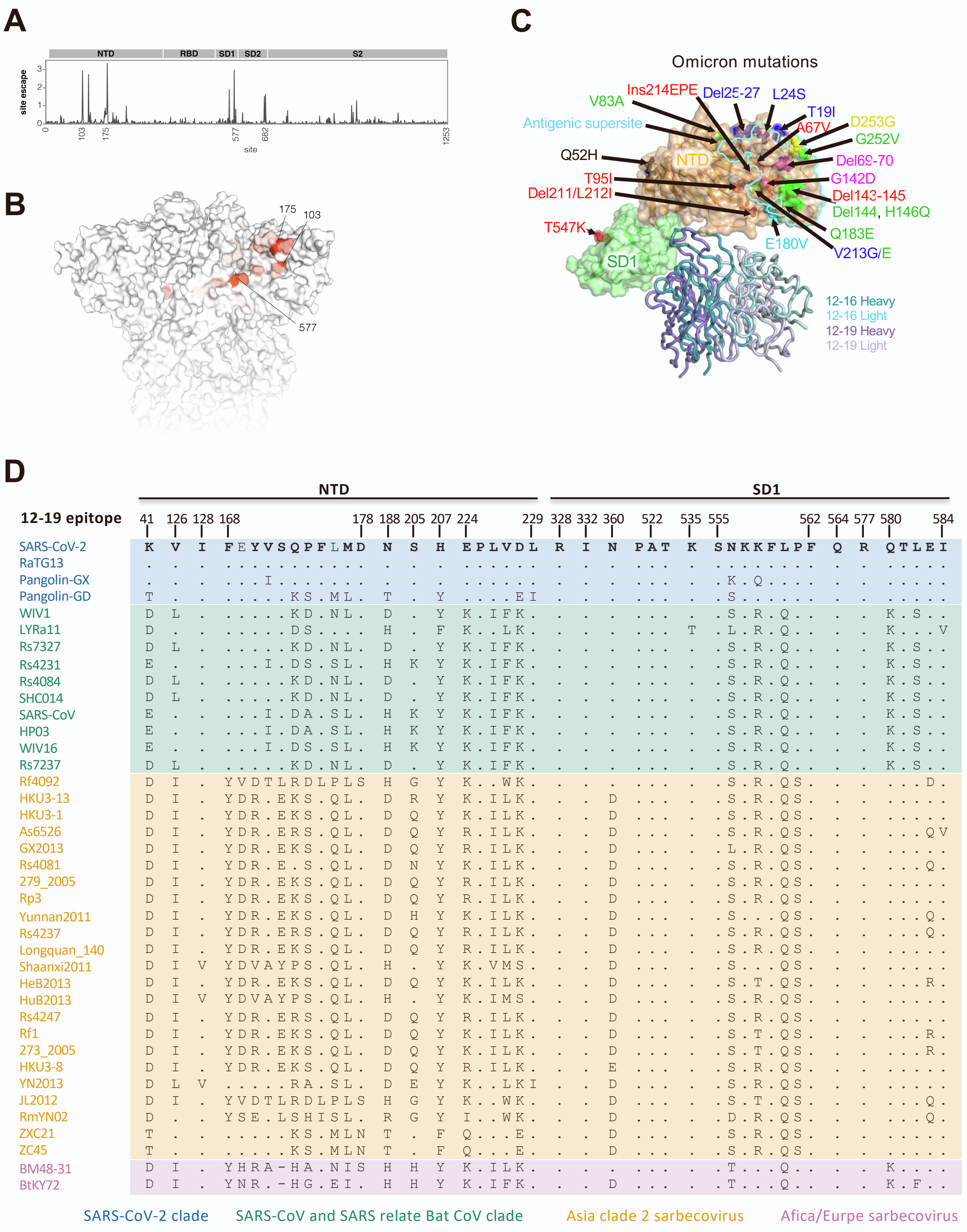

A
NTD
RBD
SD1
SD2
S2
site escape
0
1
2
3
0
103
175
577
682
1253
site
B
175
103
577
C
Omicron mutations
V83A
Ins214EPE
Del25-27
L24S
T19I
A67V
D253G
G252V
Antigenic supersite
Q52H
NTD
Del69-70
T95I
G142D
Del211/L212I
Del143-145
Del144, H146Q
T547K
Q183E
E180V
V213G/E
SD1
12-16 Heavy
12-16 Light
12-19 Heavy
12-19 Light
D
NTD
SD1
12-19 epitope
41 126 128 168 178 188 205 207 224 229 328 332 360 522 535 555 562 564 577 580 584
SARS-CoV-2
RaTG13
Pangolin-GX
Pangolin-GD
WIV1
LYRa11
Rs7327
Rs4231
Rs4084
SHC014
SARS-CoV
HP03
WIV16
Rs7237
Rf4092
HKU3-13
HKU3-1
As6526
GX2013
Rs4081
279_2005
Rp3
Yunnan2011
Rs4237
Longquan_140
Shaanxi2011
HeB2013
HuB2013
Rs4247
Rf1
273_2005
HKU3-8
YN2013
JL2012
RmYN02
ZXC21
ZC45
BM48-31
BtKY72
SARS-CoV-2 clade
SARS-CoV and SARS relate Bat CoV clade
Asia clade 2 sarbecovirus
Afica/Eurpe sarbecovirus

Figure S6. Key spike escape mutations of 12-19 identified by deep mutational scanning and sequence alignment of the 12-19 epitope across selected sarbecoviruses.

(A) Mean escape scores for 12-19 antibody at each site in the BA.1 spike.

(B) Surface representation of spike colored by mean of escape scores at that site. PDB ID: 6XR8. Site numbering is based on the Wuhan-Hu-1 sequence.

(C) Antibodies 12-16 and 12-19 in complex with NTD and SD1. The NTD antigenic supersite is outlined in cyan. The mutations observed in VOCs are denoted as spheres. Omicron BA.1, BA.2 (or BA.4/5, BQ.1), XBB.1, XBB.1.16, XBB.2.3, EG.5.1, and shared mutations in at least two variants are colored in red, blue, green, cyan, yellow, black, and magenta, respectively.

(D) The sequence positioning is aligned according to SARS-CoV-2, and dots represent the residues conserved across different viruses.

See also **Figure 6**.
